# Notes and News

**Published:** 1906-02-03

**Authors:** 


### Notes and News.

At the annual meeting of the Ladies' Guild of the London Homœopathic Hospital, held on Monday, it was stated in the report that during the year 1905 the Guild had paid over the large sum of £1,273 to the funds of the hospital, and had supplied 385 garments. There are 404 members of the Guild.

Last Saturday about 5,000 persons visited the new first-class armoured cruiser, *Black Prince*, which is being exhibited in the Victoria Dock for the benefit of the Seamen's Hospital Society, the Poplar Hospital, and the West Ham Hospital. The *Black Prince* will be on view again next Saturday afternoon and Sunday, at a small charge.

Under the direction of their Medical Officer of Health, Dr. J. F. J. Sykes, the St. Pancras Borough Council have resolved upon making a serious attempt to mitigate the ravages of consumption in that part of the Metropolis which falls within the sphere of their influence. The medical men of the district have been provided with facilities for carrying out bacteriological examinations of sputum in doubtful cases, and have been invited to notify, with the patient's consent, cases of pulmonary phthisis at the customary fee for such notifications. The Borough Council will keep a register of cases notified, and every care will be taken to treat as strictly confidential the information thus given, so that nothing may be done to prejudice the patient's occupation or employment. On the receipt of a notification the Borough Council will send by post to the patient (1) a leaflet upon how to prevent the spread of consumption, (2) a list of institutions for the segregation of advanced cases, and (3) a form for the disinfection of a room previously occupied by a consumptive. The regulations are designed both to help the individual sufferer and to protect the public, and they are so devised as to attain the greatest efficiency possible without serving in any way to oppress or harass the unfortunate victims of tuberculosis. We hope the lead given by St. Pancras will soon be followed by many other public health authorities throughout the country.

The Chelsea Hospital for Women has received £200 from its benefactress H. M. E., and £100 from "Marnie."

The Basingstoke Cottage Hospital receives £1,000 under the will of the late Sir Wyndham Spencer Portal.

The late Mr. Capper Pass, of Bristol, bequeathed £1,000 to the Bristol General Hospital, and £2,000 more conditional upon his son attaining his majority.

An anonymous donor has given £1,000 to Guy's Hospital for the purpose of endowing a bed in recognition of benefits received through a surgeon of the hospital.

Upwards of 600 guineas were voted by the Court of the Worshipful Company of Carpenters at their last meeting towards various hospitals and other charitable institutions.

Dr. William Osler, Dr. H. R. Crocker, Dr. H. H. Tooth, and Dr. T. D. Acland have been elected to fill vacancies on the Council of the Royal College of Physicians of London.

Baroness Louisa Marion von Bissing, who died last December, bequeathed £5,000, to be known as the May Lodge Trust Funds, to be applied in the erection of a home in any healthy open spot in England or Wales for poor gentlewomen suffering from cancer. Preference is to be given to wives, widows, or daughters of naval officers in reduced circumstances, such inmates to pay a small weekly sum, the object of this stipulation being to avoid the stigma of their being recipients of charity. On no account are the wives, widows, or daughters of clergymen of any denomination to become inmates of the home.

In the Wandsworth County Court last week, before his Honour Judge Arthur Russell, a case of considerable interest to medical men was heard. The plaintiff, who was the wife of a billiard-marker, brought an action against a firm of medical practitioners to recover £40 damages sustained by reason of the alleged negligence on the part of one member of the firm in diagnosing her to be suffering from typhoid fever, and thereby causing her removal to the Stockwell Fever Hospital, where she was detained for 17 days, whereas she was suffering from influenza; further, for wrongfully and negligently notifying the Wandsworth medical officer of health that she was suffering from an infectious disease, and causing her goods to be disinfected and destroyed. In the evidence it appeared that the case was an exceedingly difficult one to diagnose, and in the early stages, at any rate, there was every reason to suppose that the patient was suffering from typhoid fever; and it is not easy to see how a medical man with a proper sense of duty could have acted otherwise than the defendant in this case. The Judge took this view, and remarked that the case ought never to have been brought. He gave judgment for the defendant, with special costs.

